# *ICD-11* posttraumatic stress disorder (PTSD) and complex PTSD in a sample of prison staff: A latent profile approach

**DOI:** 10.1002/jts.23128

**Published:** 2025-01-16

**Authors:** Katie Dhingra, David Boyda, Sean M. Mitchell, Peter J. Taylor

**Affiliations:** 1 School of Humanities and Social Sciences, Leeds Beckett University, Leeds, UK; 2 Division of Psychology and Mental Health, University of Manchester, Manchester, UK; 3 School of Psychology, University of Wolverhampton, Wolverhampton, UK; 4 Department of Psychological Sciences, Texas Tech University, Lubbock, Texas, USA

## Abstract

Although empirical support for the *International Statistical Classification of Diseases and Related Health Problems* (11th ed.; *ICD-11*) distinction between posttraumatic stress disorder (PTSD) and complex PTSD (CPTSD) is growing, research into the *ICD-11* CPTSD model in prison staff is lacking. This study used latent profile analysis (LPA) to (a) determine if there are distinct groups of trauma-exposed prison governors (i.e., “wardens” in the United States and Canada) who have symptom profiles consistent with the distinction between PTSD and CPTSD and (b) identify predictors and posttraumatic maladaptive beliefs associated with the latent profiles. Trauma-exposed prison governors (*N* = 385) completed the International Trauma Questionnaire (ITQ) and a measure of traumatic life events. LPA was used to extract profiles using the six ITQ symptom clusters and revealed four profiles: *CPTSD* (8.4%), *PTSD* (14.4%), *disturbances in self-organization* (DSO; 11.0%), and *low symptoms* (66.3%). Membership in the CPTSD and DSO profiles was associated with cumulative traumatization, odds ratios (*OR*) = 1.42 and *OR* = 1.26, respectively, and poorer health, *OR* = 2.84 and *OR* = 1.64, respectively, relative to the low symptom profile, and membership in the PTSD profile was associated with younger age, *OR* = 0.91, relative to the low symptom profile. The CPTSD profile showed the highest level of posttraumatic maladaptive beliefs. This study yields empirical support for the *ICD-11* CPTSD model in prison staff. The results provide additional support for the validity of ITQ measurement of PTSD and CPTSD.

Prison or correctional officers’ are exposed to occupational violence and subjected to personal victimization and injury far more frequently than individuals in comparable occupations (Konda et al., 2012), increasing their risk for posttraumatic stress disorder (PTSD). Between 15% and 34% of prison officers worldwide meet the diagnostic threshold for PTSD (Regehr et al., 2021). However, little is known about complex PTSD (CPTSD) in prison governors (i.e., “wardens” in the United States, Canada, and other places). Although not previously examined, high rates of CPTSD are expected in prison staff, as these individuals work in environments that expose them directly and/or vicariously to prolonged and repeated traumatic experiences (King & Oliver, 2020; Slade & Lopresti, 2013). Thus, further investigation of both PTSD and CPTSD in prison governors is necessary to improve the assessment and treatment of trauma-related psychopathology.

CPTSD was included in the 11th revision of the *International Statistical Classification of Diseases and Related Health Problems* (*ICD-11*; World Health Organization [WHO], 2019) alongside PTSD under the parent category of “Disorders Specifically Associated with Stress.” CPTSD consists of six symptom clusters: reexperiencing the traumatic event in the here and now (Re), avoidance of traumatic reminders (Av), sense of current threat (Th), affective dysregulation (AD), negative self-concept (NSC), and difficulties in interpersonal relationships (DR). The first three symptom clusters are shared with PTSD, and the latter three are collectively termed *disturbances in self-organization* (DSO). These disturbances are typically associated with prolonged, repeated, or multiple forms of trauma exposure (Reed et al., 2022; WHO, 2019).

Since the publication of the *ICD-11* CPTSD description (Maercker et al., 2013), substantial support for the characterization of PTSD and CPTSD as distinct, albeit related, disorders has accumulated (Brewin et al., 2017; Redican et al., 2021). This research has been facilitated by the International Trauma Questionnaire (ITQ; Cloitre et al., 2018), which is the most commonly used self-report measure of *ICD-11* PTSD and CPTSD symptoms and diagnoses (Gelezelyte et al., 2022).

Evaluations of the ITQ in treatment-seeking and community-based samples have provided strong support for its psychometric properties (Redican et al., 2021). Further, studies have consistently reported two subgroups distinguished by different patterns of symptom endorsement: a PTSD profile and a CPTSD profile (Currier et al., 2021; Spikol et al., 2022). A systematic review of factor analytic and latent class/profile analyses (LCA/LPA) studies (Redican et al., 2021) reported that the number of profiles identified varied from two to six; however, all studies identified the presence of both a PTSD class and a CPTSD class. Most clinical studies also identified a class marked by low endorsement of both PTSD and DSO symptoms (i.e., a low symptom class), whereas an additional DSO class, characterized by prominent AD symptoms, emerged in most community studies.

In line with the proposition that CPTSD class membership is associated with higher rates of exposure to traumatic experiences and a higher number and type of clinically elevated symptoms than PTSD (Brewin et al., 2017), CPTSD class membership is associated with higher rates of traumatization (Fox et al., 2020), more psychopathology and comorbidity (e.g., major depressive disorder [MDD], dissociation, generalized anxiety disorder [GAD], behavioral problems, insomnia, emotion regulation difficulties), and higher levels of functional impairment (Haselgruber et al., 2020) than PTSD or low symptoms profiles. Demographic factors, such as younger age, living alone, and unemployment status, are also positively associated with CPTSD symptomatology (e.g., Hyland et al., 2021; Karatzias, Hyland, et al., 2019). Because CPTSD is a more severe and impairing disorder than PTSD, and scholars have argued that a different therapeutic approach to PTSD may be required to effectively treat CPTSD (Karatzias, Murphy, et al., 2019), it is important that service providers effectively differentiate between *ICD-11* PTSD and CPTSD to provide optimal treatment. Further, there is a need to continue assessing the factors uniquely associated with *ICD-11* CPTSD, as such findings can be used to guide clinical assessments, early intervention programs, and subsequent approaches to treatment.

Although previous research has highlighted the influential role of posttraumatic maladaptive beliefs (i.e., the ways in which someone thinks about themselves or the world following trauma exposure) in the development, maintenance, and treatment of PTSD (Ehlers & Clark, 2000), their association with CPTSD has been largely unexplored. Further, the association between maladaptive appraisals and PTSD/CPTSD has yet to be studied in occupational environments characterized by high rates of violence, unpredictability, and interpersonal tension, such as prisons. This is an important oversight, as the unique environment in which prison staff operate likely fosters beliefs related to threat of harm and distrust in others (Kinmann & Clements, 2020), which may increase the likelihood of CPTSD symptom presentations. Therefore, we sought to examine posttraumatic maladaptive beliefs as a distal outcome of profile membership.

To our knowledge, no studies to date have tested the *ICD-11* model of CPTSD in prison staff. Based on previous research in other trauma-exposed populations, we hypothesized that the LPA would reveal distinct profiles for CPTSD, PTSD, and low symptom presentations, as well as an additional profile, such as the DSO profile identified in previous community-based studies (e.g., Redican et al., 2021). Based on prior research, we also hypothesized that the CPTSD profile would be predicted by higher levels of trauma exposure (e.g., Cloitre et al., 2020) and associated with higher degrees of greater functional impairment (i.e., poorer subjective physical health and greater alcohol use) and posttraumatic maladaptive beliefs.

## METHOD

### Participants

All members of the Prison Governor’s Association (PGA; *N* = 1,055) were contacted by email about the study; 458 agreed to participate, but 49 were excluded from the data analysis because they did not report exposure to a potentially traumatic event (PTE). Additionally, 24 participants were excluded from the analyses due to missing data for all exogenous variables. The final sample comprised 385 prison governors who ranged in age from 26 to 75 years old (*M* = 50.10 years, *SD* = 7.71). See Table 1 for sample characteristics.

### Procedure

As noted, participants were recruited via an email distributed to all PGA members. Although it is not possible to know how many individuals saw the survey advertisement, the response rate was estimated to be 38.8%. Participants completed the study assessments anonymously on an online survey platform using a secure weblink (see Dhingra et al., 2021 for further methodological details). Participants provided informed consent, and participation was voluntary. Participants were not paid for participating. A debriefing statement and mental health service contacts were provided at the end of the survey. The ethical review board of Leeds Beckett University approved the study procedures.

### Measures

#### Demographic information, alcohol consumption, and current health

A demographic and history questionnaire (DHQ) was administered to assess demographic information (age, sex, sexual orientation, race, relationship status, employment details). The DHQ also included a single item about weekly alcohol consumption (“Do you drink alcohol?” [14 units = 6 pints of beer, 10 small glasses of wine, 14 single measures of spirits]’’), with response options ranging from 1 (*no*) to 5 (*yes, more than 21 units a week*), and another question related to the respondent’s current health (“What is your current general health state?”), with response options ranging from 1 (*very good*) to 5 (*very bad*). Descriptive statistics are reported in Table 1.

#### Lifetime trauma exposure

The Life Events Checklist for *DSM-5* (LEC-5; Weathers et al., 2013) is a 17-item self-report assessment of PTE exposure. The measure covers exposure to 16 trauma types (e.g., natural disaster, physical assault, life-threatening illness or injury), with a 17th item allowing respondents to report other stressful events not listed. For each item, participants were asked to endorse one of the following response options: “happened to me,” “witnessed it happening to somebody else,” “learned about it happening to someone close to me,” “part of my job,” “not sure it applies,” and “doesn’t apply to my experience.” For Items 1–16, participants were considered exposed to a given PTE if they reported experiencing or witnessing the event; Item 17 was excluded from the analyses due to its unspecified nature. We scored experiencing a PTE and witnessing a PTE as separate variables, each of which could range from 0–16. The LEC-5 has demonstrated good test-retest reliability, convergent validity, and significant association with PTSD symptoms (Gray et al., 2004).

#### PTSD and CPTSD symptoms and diagnosis

The International Trauma Questionnaire (ITQ; Cloitre et al., 2018) is an 18-item, self-report assessment of *ICD-11* PTSD and CPTSD. Respondents rate items on a scale of 0 (*not at all*) to 4 (*extremely*). Six items measure PTSD symptom clusters (Re, Av, and Th), six items measure DSO symptom clusters (AD, NSC, and DR), and six items measure functional impairment (i.e., social, occupational, and other important areas of life) related to the PTSD and DSO symptoms. For PTSD items, respondents are asked to consider their most distressing traumatic event and how much they have been “bothered by that problem in the past month.” For DSO items, they are asked to reflect on how they “typically” feel, think about themselves, and relate to others. The reliability and validity of the ITQ have been supported in several populations, including refugees, trauma samples, and adolescents and young adults exposed to mass shootings (Redican et al., 2021).

Dimensional scoring was used for each symptom to create the LPA indicators by summing the two items pertaining to each PTSD and CPTSD feature. This produced six continuous LPA indicators: Re, Av, Th, AD, NSC, and DR. This is consistent with previous research using the ITQ dimensional scoring for LPA indicators (e.g., Currier et al., 2021). Additionally, dimensional scoring does not rely on the endorsement of an item with a score of 2 or higher, which is not a validated criterion for the population of interest in the current study. In the present sample, Cronbach’s alpha values for each of the PTSD and DSO symptom clusters were .81 for Re, .87 for Av, .77 for Th, .50 for AD, .92 for NSC, and .86 for DR.

#### Posttraumatic maladaptive beliefs

The Posttraumatic Maladaptive Beliefs Scale (PMBS; Vogt et al., 2012) is a 15-item, self-report measure of maladaptive beliefs following trauma exposure consisting of three subscales: Threat of Harm (five items), Self-Worth and Judgement (five items), and Reliability and Trustworthiness of Others (five items). Participants were asked to rate each item on a 7-point scale ranging from 1 (*not true*) to 7 *completely true*). After reverse-scoring relevant items, scores were summed for each subscale (range: 5–35). The PMBS has shown strong content validity, internal consistency, and convergent and discriminant validity (Vogt et al., 2012). We used the subscale scores as covariates, with Cronbach’s alpha values of .78 for Threat of Harm, .72 for Self-Worth and Judgement, and .81 for Reliability and Trustworthiness.

### Data analysis

Analyses were conducted with *Mplus* (Version 8.10; Muthén & Muthén, 2017) using robust maximum likelihood estimation (Yuan & Bentler, 2000). A total of 92.8% of the data were available. Missing data were estimated using full information maximum likelihood (FIML; Schafer & Graham, 2002). A three-step LPA (Asparouhov & Muthén, 2014) was conducted using six continuous scores for PTSD (Re, Av, Th) and CPTSD (AD, NSC, DR) features. LPA aims to identify homogenous groups of individuals with similar patterns on the indicator variables. A series of one-profile to six-profile models was estimated, and optimal model fit was assessed using several indices. In addition to parsimony consideration, the Akaike information criterion (AIC; Akaike, 1987), Bayesian information criterion (BIC; Schwarz, 1978), and sample size-adjusted BIC (ssaBIC: Sclove, 1987) were used, with lower values considered indicative of a better model fit. The adjusted Lo–Mendell–Rubin likelihood ratio test (aLRT; Lo et al., 2001) and bootstrapped likelihood ratio test (BLRT; Arminger et al., 1999; McLachlan & Peel, 2000) were also applied; *p* values for the aLRT and BLRT indicate whether a solution with more profiles (*p* < .05) or fewer profiles (*p* > .05) better fits the data. Entropy values closer to 1.0 suggest more distinct profiles.

Predictors of profile membership were assessed using the auxiliary command (R3STEP) including age, sex (coded 0 = women, 1 = men), weekly alcohol consumption, health status, and LEC-5 score (experienced or witnessed a potentially traumatic event). Distal outcomes were assessed using the manual Bolck, Croon, and Hagenaars (BCH) method (Asparouhov & Muthén, 2014; Lanza et al., 2013), including the PMBS subscales (i.e., Threat of Harm, Self-Worth and Judgement, and Reliability and Trustworthiness of Others).

Post hoc power analysis using G*Power (Faul et al. 2007) showed that the power to detect sex differences in trauma-related outcomes was 53.1%. As this is below the commonly accepted threshold of 80%, and the study was not designed to examine sex differences (primarily given this is a male-dominated occupation), we did not explore potential sex differences. A second G*Power analysis was conducted to ensure sufficient power to detect differences between profiles. With four groups (low symptom, PTSD, CPTSD, DSO) and four covariates (age, sex, weekly alcohol consumption, health status), we tested for medium effect sizes *f* = 0.25) at a significance level of *α* = 0.05. Results indicated that our study had 94% power to detect differences in distal outcomes across the four latent profiles.

## RESULTS

### Descriptive statistics

Among the 385 participants, data were missing on 3.6% of the LPA indicators, 0.6% of the predictors of profile membership, and 7.1% of the distal outcomes. Participants reported experiencing an average of 2.94 (*SD* = 2.11, range: 0–9) lifetime PTEs. Participants also reported witnessing an average of 4.97 (*SD* = 3.10) lifetime traumatic events, ranging from 0 to 14. See Table 2 for descriptive statistics for all indicators, predictors, and distal outcomes variables.

### LPA

As seen in Table 3, the fit indices indicated that the four-profile solution was a better fit for the data than the one-, two-, and three-profile models given smaller AIC and BIC values and a higher entropy value. The insignificant LRT (*p* > .05) in the five-profile solution indicated that the previous four-profile was the most parsimonious solution. Model 5 was rejected on the basis that the profile size was too small to be of substantive value (i.e., less than 20), along with insignificant aLRT values and a lower entropy value. Model 6 was a poor-fitting model overall.

**FIGURE 1:**
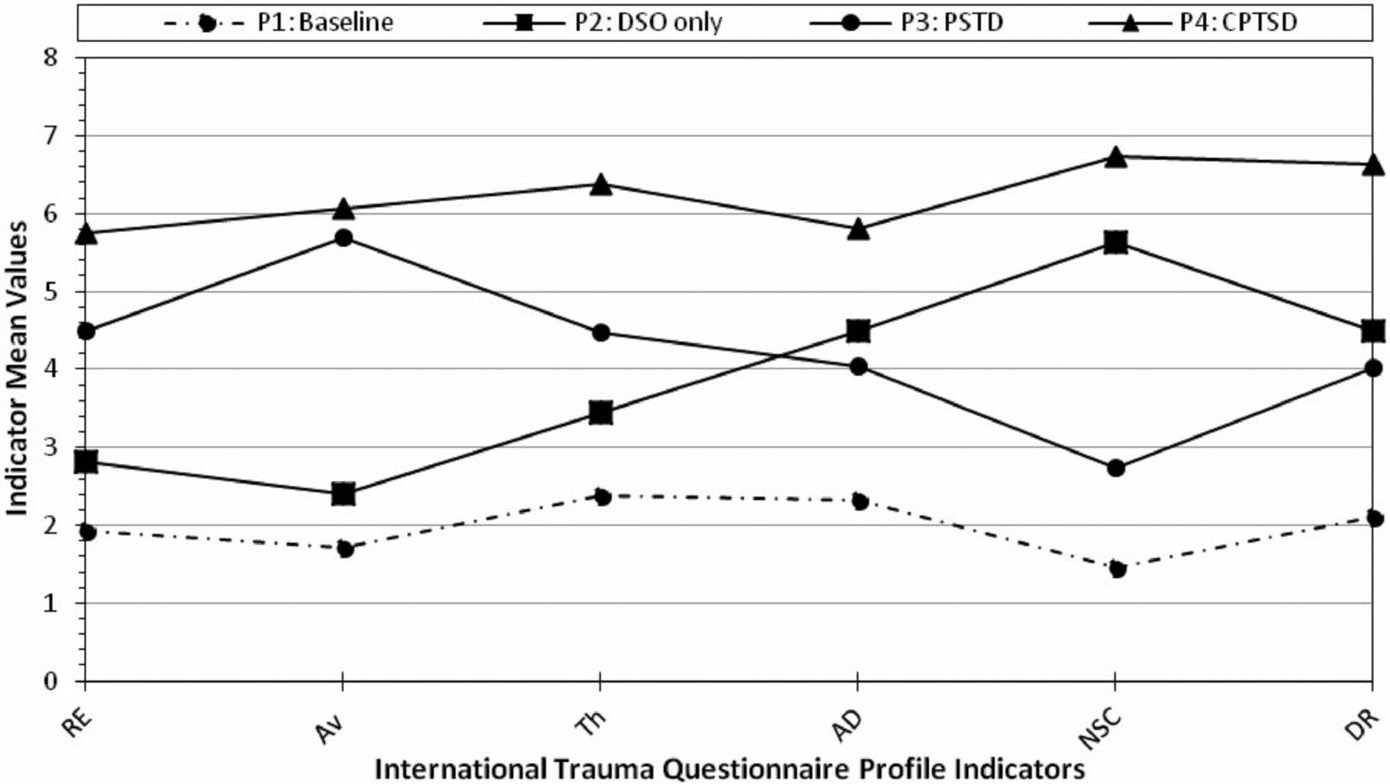
Four profile plots of trauma-related syndromes based on the International Trauma Questionnaire (ITQ) *Note*: Continuous latent profile analysis indicators included ITQ symptoms of reexperiencing in the here and now (Re), avoidance (Av), sense of current threat (Th), affective dysregulation (AD), negative self-concept (NSC), and disturbances in relationships (DR). Profile (P) 1: Baseline/low symptoms, P2: DSO = disturbances in self-organization, P3: posttraumatic stress disorder (PTSD), P4: complex PTSD (CPTSD).

Figure 1 shows the four-profile solution. Profile 1 comprised 66.3% of the sample (*n* = 258) and was labeled the *low symptom* profile; individuals in this profile endorsed the lowest scores across all LPA indicators. Profile 2, labeled *DSO*, comprised 11.0% of the sample (*n* = 42) and included individuals with moderately high AD, NSC, and DR scores. Similarly, Profile 3, labeled *PTSD*, comprised 14.4% of the sample (*n* = 52) and included individuals who endorsed elevated scores on the PTSD indicators but not the DSO indicators. Profile 4, labeled *CPTSD*, was the smallest profile (*n* = 33, 8.4%) and included individuals who endorsed the highest scores across all LPA indicators. The average probabilities for the four-profile solution ranged from .93 to .97, indicating good classification accuracy.

### Predictors of profile group membership

We conducted multinomial logistic regression to examine predictors of profile membership (see Table 4). The low symptom profile was the reference group. Older age decreased the odds of membership in the PTSD profile compared to the low symptom profile, odds ratio (*OR*) = 0.91, 95% CI [0.86, 0.98]. Poorer self-reported health, *OR* = 1.64, 95% CI [1.02, 2.66], and exposure to a higher number of trauma types, *OR* = 1.26, 95% CI [1.08, 1.49], were associated with higher odds of membership in the DSO profile compared to the low symptom profile. Poorer self-reported health, *OR* = 2.84, 95% CI [1.19, 1.81], and exposure to a higher number of traumatic events, *OR* = 1.42, 95% CI [1.19, 1.71], were associated with higher odds of belonging to the CPTSD profile compared to the low symptom profile. Sex, weekly alcohol consumption, and witnessing a higher number of traumatic events did not significantly distinguish between low symptom profile membership and membership in the other profiles.

### Distal outcomes

As seen in Table 5, individuals in the CPTD profile scored significantly higher than those in the PTSD profile on all three PMB subscales: Threat of Harm, *p* < .001; Self-Worth and Judgement, *p* < .001; and Reliability and Trustworthiness of Others, *p* = .033. Individuals in the DSO profile scored significantly higher on the PMB Self-Worth and Judgement subscale compared to those in the PTSD profile, *p* < .001; however, their scores were not statistically different from those in the CPTSD profile, *p* = .278. There was no significant difference between the DSO and CPTSD profiles for the Threat of Harm, *p* = .530, or Reliability and Trustworthiness of Others, *p* = .946, subscales.

## DISCUSSION

This study aimed to determine if the naturally occurring symptom distribution in prison governors was consistent with the *ICD-11* PTSD and CPTSD specifications. We hypothesized that the LPA would reveal distinct participant groups reflecting *ICD-11* symptom patterns for PTSD and CPTSD. We also investigated the roles of various demographic and trauma-related factors in predicting latent profile membership and explored whether posttraumatic maladaptive cognitions were associated with profile membership. We hypothesized that differences in predictors and posttraumatic maladaptive cognitions would support a potential distinction between PTSD and CPTSD.

The LPA resulted in a four-profile model that included a CPTSD profile (8.5%) characterized by high PTSD and DSO symptom levels; a DSO profile (11.0%); a PTSD profile (14.4%); and a low symptom profile (66.3%). Our observation of four distinct symptom profiles is consistent with findings from other general population studies that have identified PTSD, CPTSD, DSO, and low symptom classes or profiles (see Redican et al., 2021). These findings provide support for the *ICD-11* predictions that there are distinct trauma groups in the population, including prison governors.

Regarding the DSO profile, various proposals have been advanced about what this profile may represent, including that it (a) includes individuals with other psychological disorders not measured or yet understood (Cloitre et al., 2020; Knefel et al., 2015); (b) encompasses individuals with nonpathological or subthreshold CPTSD who are more vulnerable to DSO symptoms but more resilient to PTSD symptoms (Perkonigg et al., 2016); and/or (c) is an artifact of larger sample sizes, which can lead to identifying additional classes or profiles. Further research is needed to clarify the nature of the DSO profile (Cloitre et al., 2020). Notably, in this sample of prison governors, exposure to a higher number of trauma types and poorer self-reported health predicted membership in the DSO profile compared to the low symptom profile. This suggests a need for strategies to effectively manage and treat elevated DSO symptoms in the absence of co-occurring PTSD symptoms.

Consistent with previous studies (Ben-Ezra et al., 2018; Karatzias, Hyland, et al., 2019), PTSD profile membership was associated with younger age compared to the low symptom profile. Age did not, however, predict membership in the CPTSD or DSO profiles compared to low symptom profile membership. This may be because prison staff more affected by trauma are likely to leave the profession earlier or remain in frontline roles, making them less likely or ineligible to participate in this study, respectively. However, this finding could inform strategies to better support younger prison staff, perhaps through early intervention programs or more comprehensive training on coping with trauma. Sex was also unrelated to profile membership, which contrasts previous research showing that PTSD and CPTSD are more common in women than in men (e.g., Hyland et al., 2017; Knefel et al., 2015).

Similar to previous studies (Karatzias, Hyland, et al., 2019; Zerach et al., 2019), participants in the CPTSD and DSO groups reported poorer subjective health compared to those in the low symptom group. This further indicates that the detrimental effects of CPTSD are not limited to mental health and social functioning. Alcohol consumption was unrelated to profile membership but was relatively high across all profiles. Regarding trauma history, cumulative direct trauma exposure (i.e., LEC-5 “happened to me” response) significantly predicted membership in the CPTSD and DSO profiles compared to the low symptom profile. This finding aligns with the literature suggesting that CPTSD is a disorder that results from extreme, repeated, and prolonged traumatic experiences (Cloitre et al., 2009).

The current study further explored differences in maladaptive posttraumatic beliefs across profiles. As expected, individuals in the CPTSD profile reported higher maladaptive beliefs across all three PMBS subscales, providing further evidence that CPTSD is associated with more profound disruptions in individuals’ sense of self and worldview than PTSD (Cloitre et al., 2020). Although this is an important finding, the cross-sectional nature of our data limits conclusions about the directionality of these associations, leaving the specific role of maladaptive beliefs in profile membership unclear. This is, therefore, an important direction for future research. However, the data suggest that CPTSD group membership is associated with cognitive processes similar to those observed in PTSD.

Most participants (66.3%) were members of the low symptoms group, which was unexpected given the high level of exposure to traumatic events. The reasons for this are unclear, but one possibility is that they possess more effective coping strategies than frontline prison staff, either due to innate resilience, screening processes during recruitment for the role, or specialized training. Additionally, strong professional identity and clear role definition might confer some protection against the effects of trauma exposure. Alternatively, prison governors may have support systems that help them effectively manage trauma exposure. This finding suggests an important direction for future research.

The current findings provide further empirical support for the *ICD-11* distinction between PTSD and CPTSD by showing that trauma-exposed prison staff can be categorized into distinct profiles that align with these diagnoses. This confirms the *ICD-11* framework is a useful model for understanding trauma-related disorders in this population and could lead to more nuanced screening and diagnostic practices. Specifically, the additional empirical support for *ICD-11* CPTSD provided by this study should encourage health care professionals to screen prison staff for DSO symptoms (i.e., symptoms related to problems with affect regulation, self-identity, and relationships with others) as well as PTSD symptoms, which may help affected individuals access appropriate and timely interventions. The importance of addressing these symptoms is under-scored by associations between DSO profile membership and poorer health status when compared to low symptom profile membership, as well as higher levels of posttraumatic maladaptive beliefs (Self-Worth and Judgment) when compared with PTSD profile membership.

The findings also emphasize the need for interventions specifically tailored to address symptoms that align specifically with CPTSD symptoms as well as with comorbid conditions (i.e., poorer self-reported health) rather than relying on existing approaches to PTSD treatment. For example, interventions for individuals with CPTSD may need to focus on addressing DSO symptoms, such as emotional regulation and negative self-beliefs, in addition to traditional PTSD treatment. New, flexible modular or component-based treatment approaches for CPTSD that include patient–therapist collaboration in selecting a set of empirically supported treatment modules have been suggested (for a review, see Karatzias & Cloitre, 2019). Future studies should investigate how to optimize treatment outcomes for trauma-exposed prison staff.

The long-term impact of working in a prison, which can include regular exposure to traumatic events, is largely neglected within academic, policy, and practice discussions (Woodall, 2013). This study’s findings could inform policies that emphasize early intervention strategies. A trauma-informed approach may also help shift workplace culture, where social norms discourage displaying certain emotions or seeking support (Crawley, 2004), by reducing stigma around seeking mental health support (French, 2015).

Several study limitations are worth noting. First, although the LPA identified clinically meaningful and distinct symptom profiles, it is unclear how these findings would generalize to predominantly female samples and samples with higher overall symptom burdens. Second, there are limitations associated with the trauma history measure used (i.e., the LEC-5), which prohibited the examination of the frequency, intensity, or chronicity of trauma exposure. Further, the measure did not assess traumatic experiences specifically related to the prison environment. Future research should employ more comprehensive trauma exposure screening measures. Third, reliance on self-report data may have introduced bias, though clinician-administered assessments also present challenges (Hyland & Shevlin, 2024). Replication with alternative measurement methods is recommended. Fourth, the cross-sectional design precludes us from determining whether sociodemographic factors and maladaptive beliefs are causes or effects—or perhaps both—of CPTSD. Longitudinal research is needed to clarify these associations. Fifth, the scale scores for the two items measuring AD lacked acceptable internal reliability (Cronbach’s *α* = .50), perhaps due to reflecting opposing emotion regulation strategies (i.e., hypo- and hyperaffective responses) and individuals’ potential reliance on one of these strategies (Sele et al., 2020). This suggests that AD may be better captured by two correlated latent factors rather than one (Ben-Ezra et al., 2018), highlighting a need for refinement. Sixth, no specific measures were implemented to assess the quality of data collected (e.g., attention checks, indicators of participant engagement). However, the controlled email distribution method lends confidence that responses came from genuine participants. Finally, we did not consider diagnostic comorbidities (e.g., depression, anxiety, substance abuse). Future research should incorporate a wider range of potential correlates.

Notwithstanding its limitations, this is the first study to use LPA to demonstrate that the distinction in *ICD-11* between PTSD and CPTSD is clinically relevant in a sample of prison staff. Our findings support the *ICD-11* model of CPTSD by (a) identifying distinct groups, or profiles, of PTSD and CPTSD and (b) providing evidence that CPTSD is associated with differential predictors and outcomes when compared to PTSD. Further, these results add to the growing body of research establishing the ITQ as a valid measure of PTSD and CPTSD (Redican et al., 2021), which this study now extends to prison staff.

## Figures and Tables

**TABLE 1:** Demographic characteristics of the sample

Variable	*n*	%
Sex		
Men	278	72.2
Women	97	25.2
Sexual orientation		
Straight/heterosexual	363	94.3
Gay/lesbian	13	3.4
Bisexual	2	0.5
Note specified	6	1.6
Race		
White	361	93.8
Black	7	1.8
Asian	6	1.6
Multiracial	9	2.3
Relationship status		
Single	14	3.6
In a relationship	55	14.3
Divorced/separated	20	5.2
Widowed	5	1.3
Married	291	75.6
Work establishment type		
Women’s prison	30	7.8
Men’s prison	231	60
Male YOI	28	7.3
Mixed male adult/YOI	82	21.3
Other^a^ (e.g., secure hospital, IRC)	12	3.1
Work establishment security category^b^		
A	42	10.9
B	131	34
C	148	38.4
D	27	7
Other^a^	22	5.7
Current job grade		
Custodial manager	2	0.5
Governor	375	97.4
Other	8	2.1
Work establishment country		
England	365	94.8
Wales	11	2.9
Scotland	7	1.8
Northern Ireland	2	0.5
Self-reported current health status		
Very good	54	14
Good	179	46.5
satisfactory	138	35.8
bad	14	3.6
Self-reported alcohol use (units/week)		
None	57	14.8
< 5	108	28.1
5–14	127	33
15–21	55	14.3
> 21	38	9.9

*Note*. *N* = 385. Percentages may not sum to 100.0% due to a small amount of missing data for some variables. YOI = youth offender institution; IRC = immigration removal center.

a Examples: secure hospital, IRC.

b Adult prisoners may be held in one of four security categories. Category A: Prisoners whose escape would be highly dangerous to the public, the police, and/or the security of the state and for whom the aim must be to make escape impossible; Category B: Prisoners for whom the very highest conditions of security are not necessary but for whom escape must be made very difficult; Category C: Prisoners who cannot be trusted in open conditions but who do not have the resources and will to make a determined escape attempt; Category D: Prisoners who present a low risk, can reasonably be trusted in open conditions, and for whom open conditions are appropriate.

**TABLE 2:** Correlations and descriptive statistics

Variable	1.	2.	3.	4.	5.	6.	7.	8.	9.	10.	11.	12.	13.	14.	15.
1. ITQ Re	–	.58**	.52**	.42**	.46**	.32**	.04	.06	.11*	.20**	.23**	.05	.34**	.32**	.27**
2. ITQ Av		–	.49**	.45**	.46**	.48**	.05	−.01	.10	.18**	.22**	.11*	.39**	.36**	.30**
3. ITQ Th			–	.48**	.45**	.42**	.01	.10	.08	.19**	.26**	.03	.46**	.27**	.33**
4. ITQ AD				–	.57**	.66**	−.08	.10	.10	.23**	.23**	.17**	.53**	.46**	.45**
5. ITQ NSC					–	.60**	−.06	−.04	−.02	.25**	.20**	.14**	.50**	.57**	40**
6. ITQ DR						–	−.13*	.08	.01	.19**	.19**	.13*	.56**	.53**	.56**
7. Age							–	.27**	.02	.06	.05	−.12*	−.10	−.07	−.02
8. Sex^a^								–	.12*	.06	.14**	.12*	.05	.02	.08
9. Self-reported alcohol use									–	.15**	.03	.04	.03	.09	.04
10. Self-reported health status										–	.12*	.12*	.25**	.25**	.24**
11. LEC experienced trauma											–	.15**	.14**	.08	.16**
12. LEC-5 witnessed trauma												–	.13*	.13*	.11*
13. PMBS Threat of Harm													–	.55**	.60**
14. PMBS Self-Worth/Judgement														–	.58**
15. PMBS Reliability/Trustworthiness of Others															–
*M*	1.72	1.73	2.14	2.11	1.55	2.03	50.10		2.76	2.29	2.94	4.97	13.30	12.82	13.74
*SD*	1.96	2.08	2.13	1.82	2.06	2.17	7.71		1.17	0.75	2.11	3.10	5.64	5.19	6.01
Range	0–8	0–8	0–8	0–7	0–8	0–8	26–75		1–5	1–4	0–9	0–14	5–32	5–29	5–33

*Note*: ITQ = International Trauma Questionnaire; Re = reexperiencing in the here and now; Av = avoidance; Th = sense of current threat; AD = affective dysregulation; NSC = negative self-concept; DR = disturbances in relationships; LEC = Life Events Checklist for *DSM-5*; PMBS = Posttraumatic Maladaptive Beliefs Scale.

a Coded as 0 = women, 1 = men.

* *p* < .05.

** *p* < .01.

**TABLE 3:** Model fit indices for emergent profiles

Number of profiles	AIC	BIC	saBIC	aLRT	BLRT	Entropy
1	9,498.78	9,546.22	9,508.15			
2	8,770.53	8,845.64	8,785.36	724.86**	742.25**	.91
3	8,645.33	8,748.12	8,665.62	135.94	139.20**	.85
**4**	**8,484.02**	**8,614.48**	**8,509.77**	**171.20** **	**175.31** **	**.92**
5	8,426.51	8,584.64	8,457.72	69.84	71.52**	.84
6	8,377.99	8,563.80	8,414.67	61.05	62.51**	.88

*Note*: Bolding indicates the best-fitting latent profile solution. AIC = Akaike information criterion; BIC = Bayesian information criterion; saBIC = sample-adjusted BIC; aLRT = adjusted Lo–Mendel–Rubin likelihood ratio test; BLRT = bootstrap likelihood ratio test;

* *p* < .05.

** *p* < .01.

**TABLE 4:** Multinomial logistic regression results of the predictors of profile membership

Predictor variable	Profile membership								
	DSO			PTSD			CPTSD		
	*OR*	*SE*	95% CI	*OR*	*SE*	95% CI	*OR*	*SE*	95% CI
Age	0.97	0.02	[0.93, 1.03]	0.91**	0.03	[0.86, 0.98]	0.96	0.02	[0.91, 1.01]
Sex^a^	0.98	0.50	[0.36, 2.70]	2.10	1.07	[0.75, 5.71]	1.9	0.89	[0.74, 4.80]
Self-reported alcohol use	1.14	0.02	[0.88, 1.49]	0.65	0.16	[0.40, 1.08]	1.11	0.19	[0.79, 1.58]
Self-reported health status	1.64**	0.04	[1.02, 2.66]	1.14	0.32	[0.66, 2.00]	2.84**	0.86	[1.19, 1.81]
Experienced trauma^b^	1.26**	0.10	[1.08, 1.49]	1.17	0.12	[0.96, 1.44]	1.42**	0.13	[1.19, 1.71]
Witnessed trauma^b^	1.07	0.05	[0.97, 1.19]	1.12	0.09	[0.96, 1.33]	1.04	0.06	[0.93, 1.18]

*Note*: The low symptom profile was the reference group. DSO = disturbances in self-organization; PTSD = posttraumatic stress disorder; CPTSD = complex PTSD; OR = odds ratio; CI = confidence interval.

a Coded as 0 = women, 1 = men.

b Assessed using the Life Events Checklist for *DSM-5*.

** *p* < .01.

**TABLE 5:** Profile-specific means of distal outcomes across profiles and pairwise comparisons

Outcome variable	Profile						
	DSO (P2)		PTSD (P3)		CPTSD (P4)		Pairwise comparisons
	*M*	*SE*	*M*	*SE*	*M*	*SE*	P2 vs. P3 vs. P4
PMBS Threat of Harm subscale	16.75	0.84	16.00	0.84	20.42	1.24	P2 vs. P3 P2 vs. P4* P3 vs. P4**
PMBS Self-Worth and Judgement subscale	17.59	0.76	14.50	0.63	19.00	1.40	P2 vs. P3*** P2 vs. P4 P3 vs. P4***
PMBS Reliability and Trustworthiness of Others subscale	16.34	0.91	16.42	0.90	19.52	1.13	P2 vs. P3 P2 vs. P4* P3 vs. P4*

*Note*: DSO = disturbances in self-organization; PTSD = posttraumatic stress disorder; CPTSD = complex PTSD; PMBS = Posttraumatic Maladaptive Beliefs Scale.

* *p* < .05.

** *p* < .01.

*** *p* < .001.
